# Digestive Alkaline Proteases from *Zosterisessor ophiocephalus, Raja clavata*, and *Scorpaena scrofa*: Characteristics and Application in Chitin Extraction

**DOI:** 10.4061/2011/913616

**Published:** 2011-07-18

**Authors:** Rim Nasri, Islem Younes, Imen Lassoued, Sofiane Ghorbel, Olfa Ghorbel-Bellaaj, Moncef Nasri

**Affiliations:** Laboratoire de Génie Enzymatique et de Microbiologie, Ecole Nationale d'Ingénieurs de Sfax, P.O. Box 1173, 3038 Sfax, Tunisia

## Abstract

The aim of this work was to study some biochemical characteristics of crude alkaline protease extracts from the viscera of goby (*Zosterisessor ophiocephalus*), thornback ray (*Raja clavata*), and scorpionfish (*Scorpaena scrofa*), and to investigate their applications in the deproteinization of shrimp wastes. At least four caseinolytic proteases bands were observed in zymogram of each enzyme preparation. The optimum pH for enzymatic extracts activities of *Z. ophiocephalus*, *R. clavata,* and *S. scrofa* were 8.0-9.0, 8.0, and 10.0, respectively. Interestingly, all the enzyme preparations were highly stable over a wide range of pH from 6.0 to 11.0. The optimum temperatures for enzyme activity were 50°C for *Z. ophiocephalus* and *R. clavata* and 55°C for *S. scrofa* crude alkaline proteases. Proteolytic enzymes showed high stability towards non-ionic surfactants (5% Tween 20, Tween 80, and Triton X-100). In addition, crude proteases of *S. scrofa*, *R. clavata,* and *Z. ophiocephalus* were found to be highly stable towards oxidizing agents, retaining 100%, 70%, and 66%, respectively, of their initial activity after incubation for 1 h in the presence of 1% sodium perborate. They were, however, highly affected by the anionic surfactant SDS. The crude alkaline proteases were tested for the deproteinization of shrimp waste in the preparation of chitin. All proteases were found to be effective in the deproteinization of shrimp waste. The protein removals after 3 h of hydrolysis at 45°C with an enzyme/substrate ratio (E/S) of 10 were about 76%, 76%, and 80%, for *Z. ophiocephalus*, *R. clavata*, and *S. scrofa* crude proteases, respectively. These results suggest that enzymatic deproteinization of shrimp wastes by fish endogenous alkaline proteases could be applicable to the chitin production process.

## 1. Introduction

Proteases constitute the most important group of industrial enzymes used in the world today, accounting for approximately 50% of the total industrial enzyme market [1]. They have diverse applications in a wide variety of industries such as detergent, food, pharmaceutical, leather, peptide synthesis, and for the recovery of silver from used X-ray films [2, 3]. Proteases are mainly derived from animal, plant, and microbial sources. 

Today, there is an increasing demand for fish proteolytic enzymes in food processing. Fish viscera, one of the most important by-products of fishing industry, is known to be a rich source of digestive enzymes, especially proteases that have high activity over a wide range of pH and temperature conditions [4–6] and exhibit high catalytic activity at relatively low concentration [7]. These characteristics of fish proteases have made them suitable for some interesting new applications in food-processing operations. In addition, fish enzymes could be utilized to produce bioactive peptides from fish proteins [8, 9]. Considering the specific characteristics of these enzymes, fish processing by-products are currently used for enzyme extraction. 

The most important digestive proteolytic enzymes from fish and aquatic invertebrates viscera are the aspartic protease pepsin secreted from gastric mucosa, and the serine proteases, trypsin, and chymotrypsin secreted from the pancreas, pyloric caeca, and intestine [10]. Acidic proteases from fish stomachs display high activity between pH 2.0 and 4.0, while alkaline digestive proteases, such as trypsin, are most active between pH 8.0 and 10.0. The distribution of proteinases varies, depending on species and organs. Digestive enzymes of several species of fish have been isolated from the internal organs including gastric, intestinal, and hepatopancreas [5, 9, 11–13].

Chitin, a homopolymer of *N*-acetyl-D-glucosamine residues linked by *β*-1,4 bonds, is the most abundant renewable natural resource after cellulose [14]. Chitin and its derivatives are biomolecules of a great potential, possessing versatile biological activities, demonstrating excellent biocompatibility and complete biodegradability. Therefore, they have found extensive applications in pharmacy, medicine, agriculture, food and textile industries, cosmetics, and wastewater treatment [15–17].

The main sources of raw material for the production of chitin are cuticles of various crustaceans, principally crabs and shrimps. Chitin in biomass is closely associated with proteins, inorganic compounds (such as calcium carbonate), lipids, and pigments. They all have to be quantitatively removed to achieve the high purity of chitins necessary for biological applications [18].

Conventionally, to extract chitin from crustacean shells, chemicals processing for demineralization and deproteinization have been applied. Raw materials were first treated with dilute hydrochloric acid at room temperature to remove metal salts, particularly calcium carbonate, and then with strong bases to remove proteins [18]. However, the use of these chemicals may cause a partial deacetylation of the chitin and hydrolysis of the polymer, resulting in final inconsistent physiological properties [19]. An alternative approach to these harsh chemical treatments is the use of proteolytic microorganisms [20–23] or proteolytic enzymes [24]. Bustos and Healy [25] demonstrated that chitin obtained by the deproteinization of shrimp shell waste with various proteolytic microorganism had higher molecular weights compared to chemically prepared shellfish chitin.

In the present paper, we describe the extraction and characterization of alkaline proteases from *Z. ophiocephalus*, *R. clavata*, and *S. scrofa *which are suitable in the chitin production process.

## 2. Materials and Methods

### 2.1. Reagents

Casein sodium salt from bovine milk, trichloroacetic acid (TCA), ethylene diamine tetraacetic acid (EDTA), and bovine serum albumin were purchased from Sigma Company Co. (St Louis, Mo, USA). Hydrochloric acid and Tris(hydroxymethyl)aminomethane were procured from Panreac Quimica SA (Barcelone, Spain). Sodium dodecyl sulphate (SDS), acrylamide, ammonium persulphate, *N*,*N*,*N*,*N^'^*-tetramethyl ethylenediamine (TEMED), and Coomassie Brilliant Blue R-250 were from Bio-Rad Laboratories (Mexico City, Mexico). All other reagents were of analytical grade.

### 2.2. Materials

Goby (*Z. ophiocephalus*), thornback ray (*R. clavata*), and scorpionfish (*S. scrofa*) were purchased from the fish market of Sfax City, Tunisia. The samples were packed in polyethylene bags, placed in ice with a sample/ice ratio of approximately 1 : 3 (w/w) and transported to the research laboratory within 30 minutes. After the fish were washed with water, their viscera were separated, rinsed with cold distilled water, and then stored in sealed plastic bags at −20°C until they were used for enzyme extraction.

### 2.3. Preparation of Crude Alkaline Proteases

Viscera (20 g) were separated and rinsed with distilled water, and then homogenized for 5 minutes with 20 mL of extraction buffer (10 mM Tris-HCl, pH 8.0) with the use of tissue homogenizer. The resulting preparations were centrifuged at 8500 ×g for 30 minutes at 4°C. The pellets were discarded and the supernatants were collected and then frozen at −20°C and used as crude protease extracts. All enzymatic assays were conducted within a week after extraction.

### 2.4. Polyacrylamide Gel Electrophoresis

Sodium dodecyl sulphate-polyacrylamide gel electrophoresis (SDS-PAGE) was carried out as described by Laemmli [26], using 5% (w/v) stacking and 15% (w/v) separating gels. Samples were prepared by mixing the crude enzyme extracts at 1 : 5 (v/v) ratio with distilled water containing 10 mM Tris-HCl pH 8.0, 2.5% SDS, 10% glycerol, 5% *β*-mercaptoethanol, and 0.002% bromophenol blue. The samples were heated at 100°C for 5 minutes before loading in the gel. After electrophoresis, the gel was stained with 0.25% Coomassie Brilliant Blue R-250 in 45% ethanol-10% acetic acid and destained with 5% ethanol-7.5% acetic acid.

### 2.5. Detection of Protease Activity of Enzyme Extracts by Zymography

Protease activity staining was performed on SDS-PAGE according to the method of Garcia-Carreno et al. [27] with a slight modification. The sample was not heated before loading in the gel. After electrophoresis, the gel was submerged in buffer A (100 mM of Tris-HCl buffer (pH 9.0)) containing 2.5% Triton X-100, with shaking for 1 hour to remove SDS and allow enzyme renaturation. Triton X-100 was removed by washing the gel three times with buffer A. The gel was then immersed in 100 mL of 1% (w/v) casein in buffer A for 5 minutes at 4°C, then further incubated for 10 minutes at 50°C to allow for the digestion of the protein substrate (casein) by the active enzymes. Finally, the gel was stained with 0.25% Coomassie Brilliant Blue R-250 in 45% ethanol-10% acetic acid and destained with 5% ethanol-7.5% acetic acid. The development of clear bands on the blue background of the gel indicated the presence of protease activity.

### 2.6. Protease Assay

Protease activity in the crude alkaline enzyme extracts was measured by the method described by Kembhavi et al. [28] using casein as a substrate. A 0.5-mL aliquot of the crude enzyme extract, suitably diluted, was mixed with 0.5 mL of 100 mM Tris-HCl (pH 8.0) containing 1% (w/v) casein, and incubated for 15 minutes at 50°C. The reaction was stopped by the addition of 0.5 mL of TCA 20% (w/v). The mixture was allowed to stand at room temperature for 15 minutes and then centrifuged at 10.000 ×g for 15 minutes to remove the precipitate. The acid-soluble material was estimated spectrophotometrically at 280 nm. A standard curve was generated using solutions of 0–50 mg/L tyrosine. One unit of protease activity was defined as the amount of enzyme required to liberate 1 *μ*g of tyrosine per minute under the experimental conditions used.

### 2.7. Effect of pH on Activity and Stability of Crude Alkaline Proteases

The optimum pH of the crude protease extracts was studied over a pH range of 5.0–13.0, using casein as a substrate at 50°C. For the measurement of pH stability, the crude enzyme extracts were incubated for 1 hour at 4°C in different buffers and then the residual proteolytic activities were determined under standard assay conditions. The following buffer systems were used: 100 mM sodium acetate buffer for pH 5.0-6.0, Tris-HCl buffer for pH 7.0-8.0, glycine-NaOH buffer for pH 9.0–11.0, Na_2_HPO_4_-NaOH buffer for pH 12.0, and KCl buffer for pH 13.0.

### 2.8. Effect of Temperature on Protease Activity and Stability

To investigate the effect of temperature, the activity was tested using casein as a substrate at the temperature range from 30 to 80°C in 100 mM Tris-HCl buffer, pH 8.0 for *Z. ophiocephalus* and *R. clavata* proteases, and pH 10.0 for *S. scrofa* crude alkaline proteases. Thermal stability was-examined by incubating crude enzyme extracts for 60-minutes at different temperatures from 30 to 70°C. Aliquots were withdrawn at desired time intervals to test the remaining activity at standard conditions. The nonheated crude enzyme extracts were considered as control (100%).

### 2.9. Effects of Metal Ions, NaCl Concentration, Surfactants, and Oxidizing Agents on Proteolytic Activity of Crude Enzyme Extracts

The influence of various metals ions, at a concentration of 5 mM, on enzyme activity was investigated by adding the monovalent (Na^+^ or K^+^) or divalent (Mg^2+^, Hg^2+^, Ca^2+^, Zn^2+^, Cu^2+^, Co^2+^, Ba^2+^, or Mn^2+^) metal ions to the reaction mixture. The activity of the crude enzyme extracts without any metallic ion was considered as 100%. The effect of NaCl concentrations on the activity of the alkaline crude protease extracts was studied, using casein as a substrate, by increasing NaCl concentrations in the reaction mixture.

The effects of some surfactants (Triton X-100, Tween 80, and SDS) and oxidizing agents (sodium perborate) on alkaline crude proteases stability were studied by preincubating enzymes for 1 hour at 30°C. The residual activities were measured at optimum conditions for each crude enzyme. The activity of the crude enzyme extract without any additive was taken as 100%.

### 2.10. Preparation of Shrimp Waste Powder (SWP) and Chemical Analysis

The SWP was prepared in our laboratory. Briefly, shrimp waste, collected from the marine food processing industry, was washed thoroughly with tap water and then cooked 20 minutes at 90°C. The solid material obtained was dried, minced to obtain a fine powder, and then stored in glass bottles at room temperature. The chemical composition (proteins, chitin, lipids, and ash) was determined.

The moisture and ash content were determined according to the AOAC standard methods 930.15 and 942.05, respectively, [29]. Total nitrogen content of shrimp protein hydrolysates was determined by using the Kjeldahl method. Crude protein was estimated by multiplying total nitrogen content by the factor of 6.25.

### 2.11. Deproteinization of Shrimp Wastes by Crude Alkaline Protease Extracts

Shrimp shell wastes (15 g) were mixed with 100 mM Tris-HCl buffer pH 8.0 at a ratio of 1 : 3 (w/v), minced and then cooked for 20 minutes at 90°C to inactivate endogenous enzymes. The cooked sample was then homogenized in a Moulinex blender for about 2 minutes. The pH of the mixture was adjusted to 8.0, and then the shrimp waste proteins were digested with proteolytic enzymes at 45°C using en enzyme/substrate ratio of 10/1 (unit of enzyme/mg of protein). After 3-hour incubation at 45°C, the reaction was stopped by heating the solution at 90°C during 20 minutes to inactivate enzymes. The shrimp waste protein hydrolysates were then centrifuged at 5000 ×g for 20 minutes to separate insoluble and soluble fractions. The solid phase was washed, pressed manually through four layers of gauze, and then dried for 1 hour at 60°C. The protein content was analyzed to measure the protein removal. The press cake was packed in a plastic bag and stored at −20°C until further processing.

Deproteinization percentage (%DP) was calculated by the following equation as described by Rao et al. [30]:

(1) $$\%\text{DP}=\frac{\left[\left(P_{o}\times O\right)-\left(P_{R}\times R\right)\right]\times 100}{P_{o}\times O},$$

where *P*_*O*_ and *P*_*R*_ are protein concentrations (%) before and after hydrolysis; while *O* and *R* represent the mass (grams) of original sample and hydrolyzed residue in dry weight basis, respectively.

### 2.12. Statistical Analysis

All experiments were carried out in triplicate, and average values with standard deviation errors are reported. Mean separation and significance were analyzed using the SPSS software package (SPSS, Chicago, Ill). Correlation and regression analysis were carried out using EXCEL program.

## 3. Results and Discussion

### 3.1. SDS-PAGE and Zymography of Crude Alkaline Proteases

In order to estimate the number of proteases in the alkaline crude enzyme extracts, samples were separated by SDS-PAGE, and then proteolytic activities were revealed by casein zymography activity staining. Casein zymography is a very sensitive and rapid assay method that detects nanogram of proteins, in contrast with SDS-PAGE which detects micrograms. 

**Figure 1:**
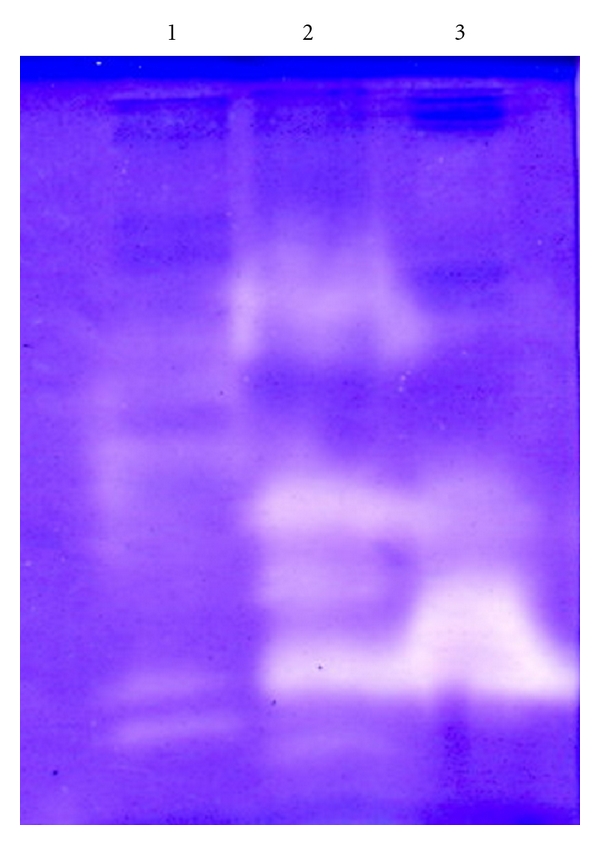
Activity staining of the crude alkaline protease extract from the viscera of *R. clavata* (1), *Z. ophiocephalus* (2), and *S. scrofa* (3).

As can be observed in Figure 1, all crude enzyme extracts showed several clear bands of protease activity with different molecular weights, indicating the presence of several different proteases. It seems that goby crude enzyme extract contained more proteolytic enzymes than the other ones as illustrated in Figure 1 by the presence of at least five clear bands of proteolytic activity. This result suggests that at least five major proteinases were present in goby viscera. When comparing the different profiles, it can be observed the presence of at least one protease common with all crude proteases. 

### 3.2. Biochemical Characterization of the Alkaline Crude Protease Extracts

#### 3.2.1. Effect of pH on Protease Activity

**Figure 2:**
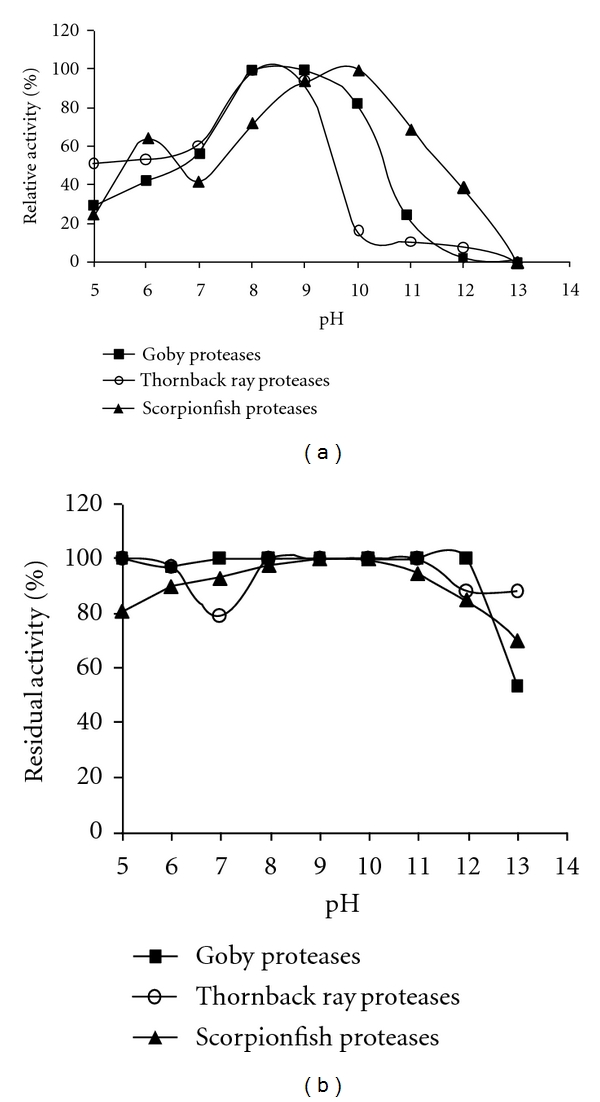
Effect of pH on activity (a) and stability (b) of alkaline crude protease extracts. The protease activity was assayed in the pH range 5.0–13.0 using buffers of different pH values at 50°C. The maximum activity of each crude enzyme extract was considered as 100%. The pH stability was determined by incubating the crude enzymes in different buffers for 1 hour at 4°C and the residual activities were measured at the optimum conditions of each enzyme preparation. The activity of the enzyme before incubation was taken as 100%. Buffer solutions used for pH activity and stability are presented in Section 2. Values are means of three independent experiments.

The activity of proteolytic enzymes was determined at different pH values from 5.0 to 13.0. The pH activity profiles of the crude alkaline proteases are shown in Figure 2(a). The proteolytic enzymes of *Z. ophiocephalus* displayed maximum activity at pH 8.0-9.0. The relative activities at pH 7.0 and 10.0 were 55.6% and 81.3%, respectively, of that at pH 9.0. However, protease activity decreased significantly above pH 10.0. At pH 11.0, the activity was approximately 5-fold lower than that at pH 9.0. 

The optimum pH for the crude protease of *R. clavata* was pH 8.0. The relative activity at pH 9.0 was about 94%. However, an appreciable decrease in activity was observed above pH 9.0.

With *S. scrofa *crude enzyme extract, two activity peaks were observed at pH 6.0 and 10.0. The enzyme preparation was highly active between pH 8.0 and 11.0, with an optimum at pH 10.0. The relative activities at pH 9.0, 11.0, and 12.0 were about 94%, 69%, and 39%, respectively, of that at pH 10.0. The optimum pH for *S. scrofa* proteases was similar to those reported by Esposito et al. [31] for proteases extracted from the viscera of *Colossoma macropomum* and El Hadj-Ali et al. [4] for proteases extracted from striped seabream (*Lithognathus mormyrus*). 

#### 3.2.2. Effect of pH on Protease Stability

The pH stability profiles of the three crude alkaline proteases are reported in Figure 2(b). Interestingly, the three crude enzyme extracts are highly stable over a wide broad pH range, maintaining about 100% of their original activity between pH 5.0 and 10.0 after 1 hour of incubation at 4°C. The enzymes retained more than 83% of their activities at pH 12.0. Our results showed that goby proteases present a high pH stability compared to the others crude enzyme extracts.

The enzyme preparation from scorpionfish, which is highly active in the alkaline pH range, was also stable over a wide pH range. These results suggest that the viscera of scorpionfish would be a potential source of alkaline proteases for certain industrial applications that require high alkaline conditions, such as detergents. In fact, one of the most important parameters for selection proteases for detergents is the optimum pH. Since the pH of laundry detergents is commonly alkaline (in the range of 9.0–12.0) [32], protease and other enzymes currently used in detergent formulations should be alkaline in nature with a high optimum pH. These properties were displayed by the scorpionfish proteases. 

#### 3.2.3. Effect of Temperature on Protease Activity

**Figure 3:**
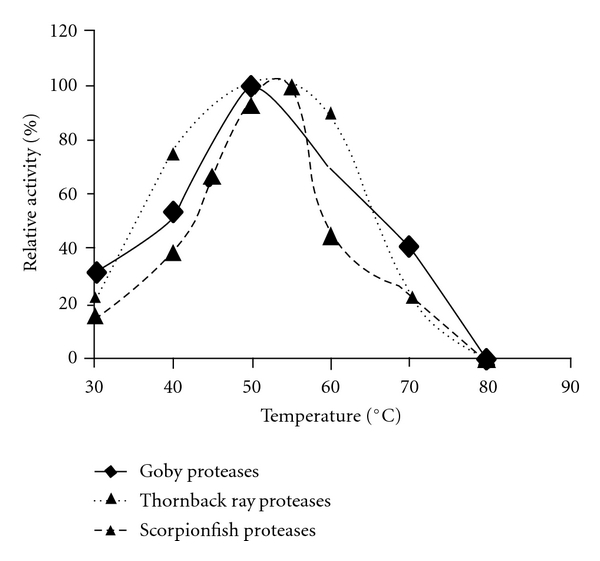
Effect of temperature on activity of alkaline crude protease extracts. The temperature profile was determined by assaying protease activity at temperatures between 30 and 80°C. The optimum activity was taken as 100%. Values are means of three independent experiments.

Optima temperatures for activity of crude alkaline proteases were determined in order to assess their suitability for biotechnological applications. The relative activities at various temperatures using casein as a substrate are reported in Figure 3. The crude proteases were active at temperatures from 30 to 70°C. The optimum temperature for *S. scrofa* proteases was 55°C, however, alkaline proteases from goby and thornback ray displayed maximum activity at 50°C. 

The relative activities of goby proteases at 40 and 60°C were 54% and 70%, respectively. However, an appreciable decrease in enzyme activity was observed above 65°C, due to thermal denaturation. Thornback ray proteases were more active at 60°C than the other crude proteases, retaining 90% of their activity after 1-hour incubation. However, the relative activities of *Z. ophiocephalus* and *R. clavata* crude proteases were 70% and 45%, respectively. 

#### 3.2.4. Effect of Temperature on Protease Stability

**Figure 4:**
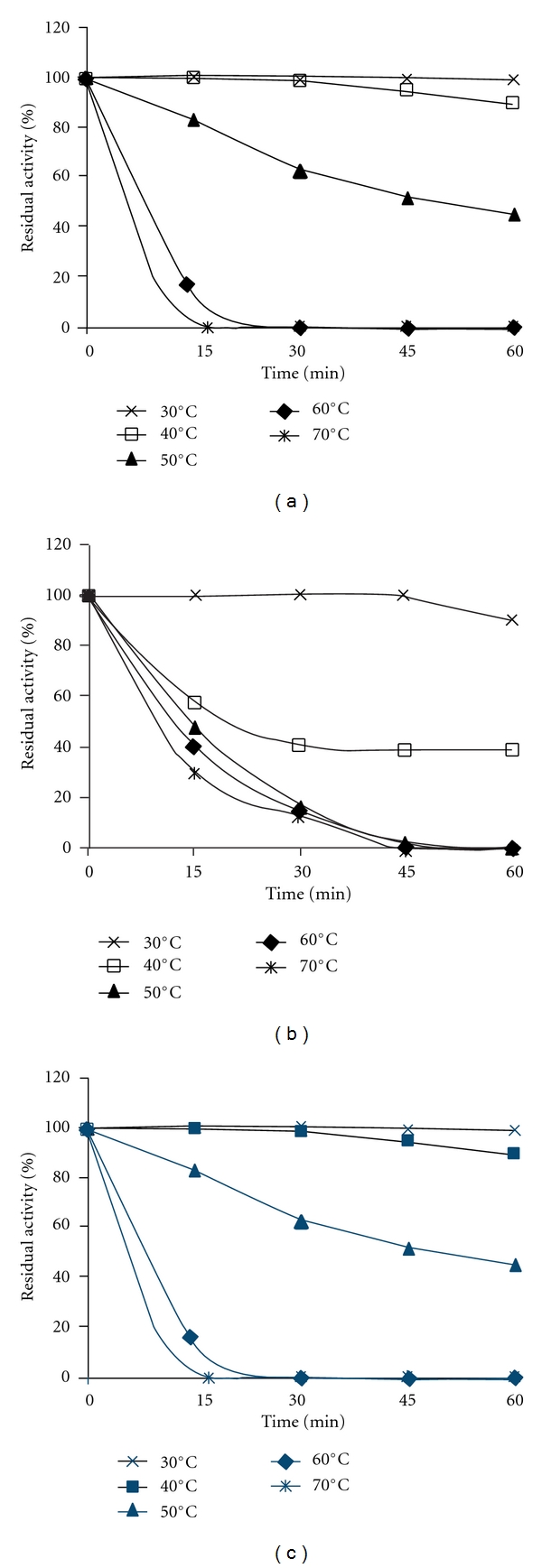
Effect of temperature on thermal stability of the crude alkaline proteases from goby (a), thornback ray (b) and scorpionfish (c). The temperature stability was determined by incubating the crude extract at temperatures from 30 to 70°C for 1 hour. The residual enzyme activity was measured under the standard conditions assay at different times. The original activity before preincubation was taken as 100%. Values are means of three independent experiments.

Thermal stability of crude alkaline proteases is depicted in Figure 4. Enzyme preparations from goby and scorpionfish are highly active at temperatures below 40°C, while that of thornback ray were stable at 30°C. Goby crude enzyme remains fully active even after 60 minutes of incubation at 40°C, indicating that this crude enzyme might be used under mild heating conditions. However, at higher temperatures proteases were inactivated.

The enzyme preparations from *Z. ophiocephalus* and *S. scrofa* retained about 24% and 45% of their initial activity after 60 minutes of incubation at 50°C, respectively. However, the proteolytic enzymes from *R. clavata* were completely inactivated in the same conditions.

#### 3.2.5. Effects of Metal Ions on Protease Activity

The effects of various metal ions, at a concentration of 5 mM, on the activity of the crude alkaline proteases were studied at optimum conditions for each crude enzyme by the addition of the respective cations to the reaction mixture (Table 1). 

The addition of CaCl_2_ and MgSO_4_ increased the activity of crude protease extracts of goby and scorpionfish. Ca^2+^ increased the activity of crude proteases from goby and scorpionfish to 110% and 129%, respectively. These results indicated that Ca^2+^ was very effective in improving the activity of the crude proteases. The enhancement of protease activity in the presence of calcium may be explained by the strength of interactions inside protein molecules and the better stabilization of enzymes against thermal stabilization. However, the activity of *R. clavata* crude enzyme was not affected by CaCl_2_.

The ions Ba^2+^ affect partially the protease activity with a relative activity between 87% and 96%. However, Fe^2+^ and Hg^2+^ affect greatly the activity of all crude enzymes. The presence of 5 mM NaCl and KCl did not affect protease activity.

#### 3.2.6. Stability of the Enzyme Extracts in the Presence of Oxidizing Agents and Surfactants

All the commercial detergents contain hydrolytic enzymes such as proteases. In addition to activity and stability at high pH range and various temperatures [33], enzymes incorporated into detergent formulations must be compatible and stable with all commonly used detergent components such as surfactants, perfumes, oxidizing agents, and other additives which might be present in the formulation [34]. Furthermore, detergent enzymes should be stable during storage and active during washing in the detergent solution for a long period of time [35].

The suitability of crude alkaline proteases as detergent additive was investigated by testing their stability in the presence of some surfactants and oxidizing agents. As shown in Table 2, crude protease extracts were highly stable in the presence of non-ionic surfactants such as Tween 20, Tween 80, and Triton X-100. Furthermore, the activities of scorptionfish and thornback ray proteases were slightly enhanced. For example, the activities of scorptionfish after incubation for 1 hour at 40°C were 107%, 109%, and 107% in the presence of 5% Triton X-100, Tween 20, and Tween 80, respectively. However, the strong anionic surfactant (SDS) at 1% caused 100% inhibition proteolytic activity of *R. clavata* proteases. 

In addition, we investigated the effects of oxidizing agents on the crude protease extract. Thornback ray and goby proteases were little influenced by oxidizing agents, retaining about 70% and 66% of their initial activity after incubation for 1 hour at 30°C in the presence of 1% (w/v) sodium perborate, respectively. 

Interestingly, the crude enzyme of scorpionfish remains fully active after 1 hour incubation at 40°C. The stability of scorpionfish enzyme extract against sodium perborate was higher than A21 protease from *Bacillus mojavensis* which retained 35% of its initial activity in the presence of 1% oxidizing agent after incubation for 1 hour at 30°C [36]. The high stability of scorpionfish enzyme extract in the presence of oxidizing agents is a very important characteristic for its eventual use in detergent formulations. Few published reports are available on the compatibility of alkaline proteases with oxidizing agents. Important commercial detergent proteases like Subtilisin Carlsberg, Subtilisin BPN, Alcalase, Esparase, and Savirase are stable in the presence of various detergent components. However, most of them are unstable in the presence of oxidant agents, such as hydrogen peroxide [34].

#### 3.2.7. Effect of NaCl

**Figure 5:**
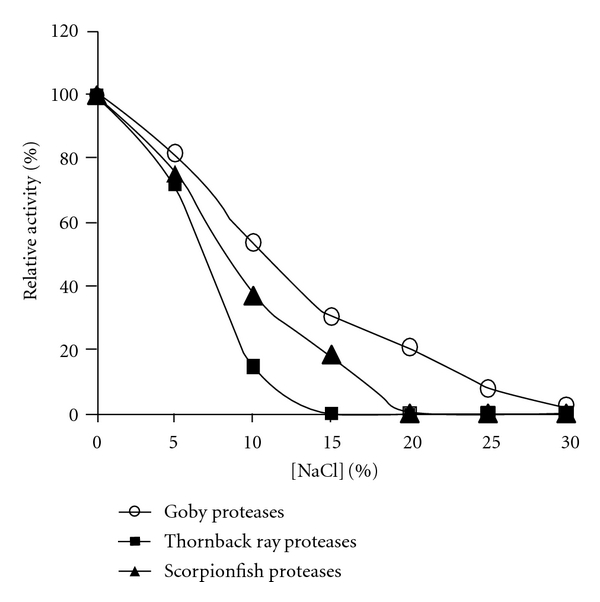
Effect of NaCl concentration on the activity of alkaline crude protease extracts.

The effect of NaCl concentration on the activity of crude alkaline proteases is shown in Figure 5. The activity of the three enzyme preparations was affected by NaCl. The activities of all crude proteases decreased gradually with increasing NaCl concentration. The relative activities of goby, scorpionfish, and thornback ray at 10% NaCl were approximately 53%, 37%, and 14%, respectively. The results showed that goby proteases exhibited a high activity in the presence of NaCl compared to the other crude enzymes. The decrease in activity might be due to denaturation of enzymes caused by the “salting out” effect with increasing NaCl concentrations.

#### 3.2.8. Enzymatic Deproteinization of Shrimp Wastes by Crude Alkaline Proteases

Chitin, a polysaccharide found in abundance in the shell of crustaceans, is closely associated with proteins. Therefore, deproteinization in chitin extraction process is crucial. Chemical treatment requires the use of HCl and NaOH, which can cause deacetylation and depolymerization of chitin. 

Few studies on the use of proteolytic enzymes for the deproteinization of shrimp wastes have been reported. To the best of our knowledge, there are no available reports on the enzymatic deproteinization of shrimp wastes by fish proteases. Many factors, such as the specificity of the enzyme used for the proteolysis, E/S ratio and the conditions used during hydrolysis (initial temperature value and hydrolysis time) have been reported to influence the enzymatic deproteinization process. 

**Figure 6:**
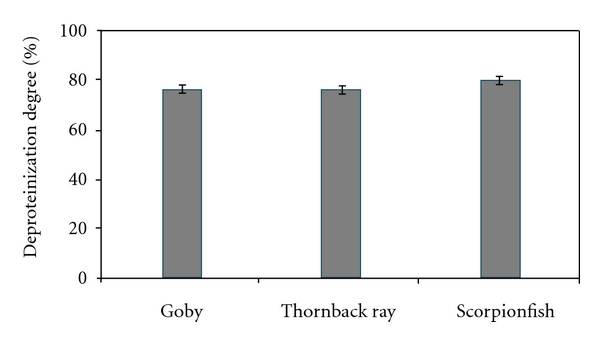
Deproteinization degree of shrimp waste by the crude alkaline proteases.

In the present study, alkaline proteases from *Z. ophiocephalus*, *R. clavata*, and *S. scrofa* were applied for the deproteinization of shrimp waste to produce chitin and protein hydrolysates using an E/S ratio of 10 U/mg. As depicted in Figure 6, all fish extracts were efficient in shrimp waste deproteinization, and *S. scrofa* crude extract was the most efficient with a deproteinization percentage of 80%. The deproteinization degrees with *Z. ophiocephalus *and *R. clavata* crude enzymes were 76%.

The deproteinization activity of crude proteases used in this study was similar to many bacterial proteases reported in many previous studies [20, 25].

## 4. Conclusion

In the present study, alkaline proteases were extracted from the viscera of *Z. ophiocephalus*, *R. clavata* and *S. scrofa *and characterized, and their efficiencies in deproteinization of shrimp waste to produce chitin were investigated.

Crude alkaline proteases from *Z. ophiocephalus*,* R. clavata*, and *S. scrofa* showed optimum activity at pH 8.0-9.0, 50°C; pH 8.0, 55°C, and pH 10.0, 55°C, respectively. The crude enzyme extract showed a high activity and stability in high alkaline pH. These proteolytic enzymes remained fully active in the presence of non-ionic surfactants. They also revealed high resistance when incubated with 1% sodium perborate. 

The alkaline crude proteases were found to be effective in the deproteinization of shrimp waste powder. The protein removals with a ratio E/S of 10 were more than 76%. 

Considering their promising properties, crude protease extracts used in this study may find potential applications in the deproteinization of shrimp waste to produce chitin and chitosan. Further research is needed to purify alkaline proteases, and to determine their properties as a possible biotechnological tool in the fish processing and food industries.

## Figures and Tables

**Table 1:** Effects of various metal ions (5 mM) on protease activity.

Metal ions	Relative activity (%)		
	Goby	Scorpionfish	Thornback ray
Control	100	100	100
Na^+^	100	91	110
K^+^	100	91	80
Mg^2+^	117	122	100
Mn^2+^	47.5	83	37.5
Zn^2+^	20	105	23
Cu^2+^	17.5	67	47.5
Hg^2+^	36	62	29.5
Fe^2+^	0	31	0
Ca^2+^	110	129	111
Ba^2+^	110	97	100

**Table 2:** Stability of alkaline proteases in the presence of various surfactants and oxidizing agents.

Surfactants/oxidizing agents	Concentration (%)	Residual activity (%)		
		Goby	Scorpionfish	Thornback ray
None	0	100	100	100
Triton X-100	5 (v/v)	100	107	117
Tween 20	5	82	109	115
Tween 80	5	90	107	100
SDS	0.1 (w/v)	40	73.5	13
	0.5	33	44	0
	1	14	16	0
Sodium perborate	0.2	92.3	106	88
	1	66	100	70

Enzyme preparations were incubated with different surfactants and oxidizing agents for 1 hour at 30°C and the remaining activity was measured under standard conditions. The activity is expressed as a percentage of the activity level in the absence of additives.
